# System and facility readiness assessment for conducting active surveillance of adverse events following immunization in Addis Ababa, Ethiopia

**DOI:** 10.1093/inthealth/ihac085

**Published:** 2023-11-03

**Authors:** Eden Dagnachew Zeleke, Getnet Yimer, Leuel Lisanework, Robert T. Chen, Wan-Ting Huang, Shu-Hua Wang, Sarah D. Bennett, Eyasu Makonnen

**Affiliations:** a Center for Innovative Drug Development and Therapeutic Trials for Africa, College of Health Sciences, Addis Ababa University, Addis Ababa, Ethiopia; b Department of Midwifery, College of Health Science, Bule Hora University, Bule-Hora, Ethiopia; c Ohio State University, Global One Health Initiative, Eastern Africa Regional Office, Addis Ababa, Ethiopia; d Brighton Collaboration, Task Force for Global Health, Decatur, GA, USA; e Department of Internal Medicine, Division of Infectious Diseases, Ohio State University, N-1120 Doan Hall, 410 West 10th Ave, Columbus, OH 43210, USA; f Ohio State University Global One Health Initiative, N-1120 Doan Hall, 410 West 10th Ave, Columbus, OH 43210, USA; g Centers for Disease Control and Prevention, 1600 Clifton Road, NE, Mailstop H24-2, Atlanta, GA 30333, USA; h Department of Pharmacology and Clinical Pharmacy, College of Health sciences, Addis Ababa University, Addis Ababa, Ethiopia

**Keywords:** active vaccination surveillance, adverse events following immunization (AEFI), readiness assessment, vaccine pharmacovigilance

## Abstract

**Background:**

To help distinguish vaccine-related adverse events following immunization (AEFI) from coincidental occurrences, active vaccine pharmacovigilance (VP) prospective surveillance programs are needed. From February to May 2021, we assessed the system and facility readiness for implementing active AEFI VP surveillance in Addis Ababa, Ethiopia.

**Methods:**

Selected hospitals were assessed using a readiness assessment tool with scoring measures. The site assessment was conducted via in-person interviews within the specific departments in each hospital. We evaluated the system readiness with a desk review of AEFI guidelines, Expanded Program for Immunization Guidelines and Ethiopian Food and Drug Administration and Ethiopian Public Health Institute websites.

**Results:**

Of the hospitals in Addis Ababa, 23.1% met the criteria for our site assessment. During the system readiness assessment, we found that essential components were in place. However, rules, regulations and proclamations pertaining to AEFI surveillance were absent. Based on the tool, the three hospitals (A, B and C) scored 60.6% (94/155), 48.3% (75/155) and 40% (62/155), respectively.

**Conclusions:**

Only one of three hospitals assessed in our evaluation scored >50% for readiness to implement active AEFI surveillance. We also identified the following areas for improvement to ensure successful implementation: training, making guidelines and reporting forms available and ensuring a system that accommodates paper-based and electronic-based recording systems.

## Introduction

National immunization programs (NIPs) deliver vaccines approved by their respective national regulatory authorities (NRAs) for their safety and effectiveness. However, because no vaccine is 100% safe and adverse events do occur during vaccination, managing adverse events following immunization (AEFI) is crucial.^1^

An AEFI can be defined as ‘any untoward medical occurrence which follows immunization and does not necessarily have a causal relationship with the usage of the vaccines’.^1^ These can be unexpected signs, symptoms, diseases or abnormal laboratory findings. AEFI are often not acceptable to the general public because of the high expectations for vaccine safety and AEFI might give vaccination opponents a reason for being against vaccination.^2^ This is especially valid when a NIP matures with the associated higher vaccination coverage, decreased incidence of targeted vaccine-preventable diseases (VPDs) and increased incidence of AEFI.^3^

Vaccine pharmacovigilance (VP) is the science and activities relating to early detection of adverse events, assessment and understanding of risk, taking appropriate action and communicating AEFI or immunization-related issues.^4^ AEFI surveillance might potentially preserve public trust if it provides reliable data on vaccine safety and communicates updated information on the benefits and risks of vaccines.^5^ This is challenging because coincidental ‘background’ events (e.g. fever) can occur in the absence of a new vaccination. Furthermore, since most AEFI lack unique laboratory or clinical features, linking AEFI to vaccine causality can be difficult. Large clinical trials or epidemiologic studies are needed to assess the differences between the rates of AEFI in vaccinated and unvaccinated individuals to evaluate for possible vaccine causality.^6^

Surveillance of immunization safety can be active or passive.^7^ Active surveillance uses actively collected data or measures outcomes to characterize the AEFI profile, rates and risk factors. Countries could achieve this through sentinel sites using selected institutions for specific AEFI and cohort event monitoring that is carried out in the community setting. Passive surveillance uses spontaneously reported data from healthcare providers, parents, caregivers and others.^8^ Unverified diagnoses, underreporting of non-serious adverse events data and biased reporting of AEFI with a close temporal link between the reported AEFI and vaccination are some of the drawbacks of passive surveillance systems. Most importantly, data collected from passive surveillance provides less than one-fourth of the scientific data needed to calculate rates of AEFI and to assess casualty.^9^

In contrast, when properly conducted, active surveillance can accurately identify and compare the rate of AEFI based on the vaccination status, thereby providing the evidence needed for timely and appropriate risk assessment and risk management response.^7^

Due to its low cost and simplicity, passive surveillance for AEFI is recommended by the World Health Organization (WHO) as the starting point for vaccine safety monitoring for any NIP and NRA.^10^ Countries with functional vaccine safety surveillance systems are expected to record at least 10 reported AEFI per 100 000 surviving infants annually.^11^ For Ethiopia this corresponds to at least 0.0868 AEFI reports per 100 000 surviving infants annually. From 2015 to 2020, however, Ethiopia only reported 3–740 AEFI, as shown in Table 1.^12^ From those 740 AEFI reported cases in 2015, 450 non-serious and 2 serious AEFI were from an integrated meningitis A and measles vaccination campaign in Afar, Amahara, Dire Dawa, Harari, Oromia and Tigray region,^13^ while all 53 AEFI reported in 2018 at the national level were detected during measles supplementary immunization activities and the human papillomavirus virus (HPV) vaccination campaign. This suggests that getting AEFI information during routine vaccination is still challenging in Ethiopia.^14^

The use of active vaccine safety surveillance is warranted when introducing a novel vaccine with limited safety data, when a well-established new vaccine is introduced to a country or if a new signal is detected through the passive safety surveillance system, warranting further investigation.^15^ While active surveillance can supplement passive surveillance, it requires more time and programmatic resources, which increase the cost.^16^ The WHO’s COVID-19 Vaccines Safety Surveillance Manual states that each country needs ‘to determine if they have the capacity to implement active surveillance of adverse events of special interest (AESI)’ and ensure the time to implement active surveillance.^17^

In Ethiopia, there are no published data on the readiness of the VP system or designated health facilities to implement active surveillance for AEFI. This evaluation aimed to assess the system and facility readiness for implementing an active AEFI surveillance in Addis Ababa, Ethiopia.

## Methods

### Study design and period

The study design was mixed method (qualitative and quantitative) and it was conducted from February to May 2021.

### Study area

The study was conducted in Addis Ababa, Ethiopia, where the estimated population in 2020 was 4 793 699, with an annual growth rate of 4.4%.^18^ There were 13 government hospitals, 32 health centres, 30 private hospitals and 7 non-governmental organization clinics in Addis Ababa during the study period.

### Site and staff selection

The three tertiary public hospitals (hospitals A, B and C) were selected based on the following criteria: ongoing collaborations with the Ethiopian Food and Drug Administration (EFDA), coverage of large populations as referral hospitals, previous experience in research and proximity to key AEFI stakeholders in Addis Ababa. The interviewees were selected because they were representative of the targeted departments.

## Data collection tool and procedure

### System readiness assessment

In the system readiness assessment, we conducted a review of AEFI guidelines, Expanded Program on Immunization (EPI) guidelines and the EFDA and Ethiopian Public Health Institute (EPHI) websites as of 23 May 2021 to understand existing vaccine safety surveillance activities (e.g. rules, proclamations and regulations; available AEFI guidelines, manuals and reporting tools; established structures and data reporting flow to facilitate reporting; communications on AEFI surveillance; and EPI support for the reporting of AEFI). This desk review included both published and unpublished documents and online sources.

### Hospital readiness assessment

A readiness assessment tool with scoring measures was used for this assessment. The tool included both closed- and open-ended questions. Scoring measures for each question assisted investigators in grading the hospital after the visit. The development of the readiness assessment tool was guided by a framework developed by Carr et al.,^19^ describing six primary dimensions of research readiness. This framework was adapted to the Ethiopian context to assess readiness for conducting active AEFI surveillance. The elements included

- Data readiness: Does the hospital have quality data recording and reporting practices?
- Record system readiness: Can the records available at the hospital capture key information needed for active AEFI surveillance?
- Organizational readiness: Is the organizational environment supportive of ‘taking on’ active AEFI surveillance?
- Study-specific readiness: Are the clinical staff knowledgeable and/or interested in conducting AEFI surveillance?
- Governance readiness: Does the study meet legal and local health system regulatory requirements?
- Business process readiness: Does the hospital have the capacity and capability to take on active AEFI surveillance?

Readiness assessment tool questions assessed the availability of AEFI guidelines and tools; awareness about the AEFI surveillance and reporting guidelines, processes and tools; data and record-keeping methods; and the willingness of staff and management to take on intensive active surveillance. To facilitate visits and interviews, the departments organized the questions, the review of facility logs and data collection tools. Questions could be repeated in more than one department (e.g. awareness of AEFI surveillance, availability of guidelines and tools) if relevant to more than one department.

Using a readiness assessment tool, investigators conducted interviews in hospitals A and B with medical directors; the heads of EPI, emergency department (both paediatrics and adult), laboratory department, pharmacy, radiology and medical records department; heads of the paediatrics department or a paediatrician; adult ward heads or a senior physician; acute flaccid paralysis (AFP) focal person(s) or surveillance office head; and the Health Management Information System (HMIS) office head. If an appropriate interviewee was not available on the day of the interview, we selected the most senior staff member available after attempting to reach the main interviewee.

Hospital C provides only maternal and neonatal care. We interviewed the heads of the neonatal intensive care unit (NICU), obstetrics and gynaecology ward, HMIS unit, ultrasound unit, EPI, laboratory department, pharmacy department, adult emergency ward, the medical director and the medical records department director. The interviewer was assisted by other departmental staff in all hospitals to review clinical records and/or other departmental materials related to AEFI reporting. In addition to observing non-identifiable clinical records, investigators used survey instruments and held discussions with key clinical and management staff in the hospital to understand reporting protocols and hospital organization; cadres of staff involved in reporting described the status of collaboration and coordination among the EFDA, EPI and EPHI. Following each site visit, investigators tallied the overall visit score to identify facility readiness to conduct active AEFI surveillance. In addition to the overall score, the facility leadership and stakeholders’ interest in participation were considered.

### Data quality control

To ensure the quality of data, the staff of the US Centers for Disease Control and Prevention (CDC) provided training on the basics of vaccine safety surveillance and the interview techniques. The overall study process was supervised and captured data and scoring were cross-checked by two investigators.

### Data analysis

The scoring measures in the readiness assessment tool were based on specific criteria (see Supplementary material). The scores for each question were added up and a percentage of the total score was calculated.

## Results

### System readiness assessment

The EFDA is responsible for ensuring the safety, quality and efficacy of medicines, including vaccines. Per regulation 299/2013, any complaints, including the safety of medicines, should be reported.^20^ There were no vaccine safety surveillance–specific proclamations or rules and regulations. According to proclamation 1112/2019, ‘Manufacturers and importers shall perform periodic monitoring of the quality, safety, and efficacy or effectiveness of its manufactured or imported medicines, perform post-marketing surveillance to establish a vigilance system, and continuously provide adverse event information’.^21^

Regulatory Standards Setting and Information Delivery (RSSID), the product safety directorate, was the designated responsible body for the collection, detection, assessment, monitoring and prevention of AEFI in the EFDA.^22^ The Ethiopian Pharmacovigilance Center is also under this directorate.

The Guidelines for Surveillance and Response to Adverse Events Following Immunization (unpublished) provide detailed information on the reportable AEFI, roles and responsibilities of stakeholders involved in AEFI surveillance, how and where to report, AEFI investigation, analysis of AEFI data, laboratory testing of specimens, AEFI causality assessment, including action and response to AEFI, and communications and media management.^23^

The recently updated implementation guidelines for EPI define AEFI and discuss AEFI classification and the need to document, report, investigate, monitor and communicate AEFI with parents, healthcare workers and the community.^24^ Additional findings from the system readiness assessment are summarized in Table 2.

### Hospital readiness assessments

Based on the tool, the three tertiary public hospitals (A, B and C) scored 60.6% (94/155), 48.3% (75/155) and 40% (62/155), respectively.

### Data and record system readiness

The data and record system readiness in participating hospitals are summarized in Table 3. Data systems in all three hospitals were largely paper based, except for the laboratory and radiology departments of hospitals A and B. These two departments used an electronic or ‘hybrid’ record system and matched the results of every child, using a unique identification or medical record number (MRN), to their medical chart. However, the main drawback of their electronic software was that it could not generate data on specific queries.

Hospital A uses a customized WHO coding system to code the diagnosis, and this is the only hospital that had incompletely applied the International Classification of Disease, Revision 9 or 10 (ICD-9/10) coding system whenever appropriate, while the other two hospitals did not use it. The ICD-9/10 coding system was used inconsistently because it involves a long, exhaustive and time-consuming list.

The physicians or paediatricians reported that during medical encounters, they did not ask children or their caregivers about recent vaccinations and most patients did not bring their vaccination record books.

**Figure 1.**
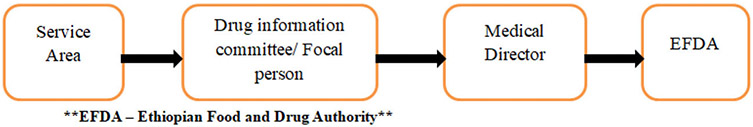
AEFI reporting flow in hospitals A and C in Addis Ababa, Ethiopia from February to May 2021.

Interviewees knew that detected AEFI should be reported to EFDA within 72 h. In addition to the reported pathway described in Figure 1, the hospital A vaccination clinic also reported AEFI to the health centre, which supplied their vaccine doses. Hospital A received feedback for some of the reported AEFI and the outcomes. The AEFI were first reported to the EFDA by hospitals A and C.

### Study-specific readiness

Four respondents from participating departments were familiar with the National Guidelines for Surveillance of AEFI—the medical director and adult emergency department head from hospital A and the facility heads of hospitals B and C. Of the participants interviewed, 5/8 (62.5%) from hospital C, 4/8 (50.0%) from hospital B and 3/8 (37%) from hospital A described AEFI ‘as an untoward/unexpected medical occurrence’; 5/8 (62.5%) participants from hospital C, 4/8 (50.0%) participants from hospital B and 5/8 (62.5%) participants from hospital A stated that ‘AEFI followed immunization’; and 2/8 (25.0%) participants from hospital B and 1/8 (12.5%) from hospital A responded that ‘it did not necessarily have a causal relationship with the vaccine’.

### Organizational readiness and business process readiness

All three hospital managements were supportive that their facilities take on more intensive AEFI surveillance. Hospital A was the only hospital to report undertaking activities, such as training and an awareness campaign, to promote AEFI reporting or improve awareness of AEFI. However, the training had only been conducted during the establishment of a VP centre in the facility.

### Governance readiness

All hospitals applied rules, regulations and guidelines (i.e. staff attendance, dress code, performance evaluation, professional conduct in clinical areas and disciplinary action, including probation, suspension, termination and expulsion of staff). All staff members were aware of the policies and about their job description. However, there were no specific rules, regulations and operational guidelines regarding AEFI surveillance in place at any of the hospitals.

## Discussion

We conducted a rapid readiness assessment for the implementation of hospital-based active sentinel site surveillance, using a tool with standardized questions and scoring. Only one of three hospitals assessed in Addis Ababa in our evaluation scored >50% for readiness to implement active AEFI surveillance. However, there has been no published benchmark (cut-off point) for measuring the readiness of facilities before implementing active surveillance; in our study, we selected the hospital with the highest score. Additionally, the desk review of the surveillance system and facility readiness assessments found an already established VP system, guidelines, reporting forms and pathways, DHIS2 (a HMIS) and the willingness of staff and administrators to conduct intensive active surveillance as possible facilitators of successful implementation of active surveillance at the hospital. However, we found overall low awareness of AEFI, the definition of AEFI, reporting mechanisms and the availability of guidelines and reporting forms. These need to be corrected to improve the chances of successful implementation. We also found that any improved AEFI surveillance activity needs to accommodate paper- and electronic-based data. If Ethiopia decides to roll out AEFI surveillance more broadly, the tool and the scoring system used for this readiness assessment could also be utilized to identify additional sites to implement AEFI surveillance.

While there are existing regulations and proclamations on reporting adverse drug reactions, there is no independently specified statement on VP in Ethiopia. In the USA, manufacturers and healthcare workers are required by regulation (21 CFR 600.80) to report all detected AEFI for any vaccine to the Vaccine Adverse Event Reporting System.^25^ The contents of Ethiopia’s AEFI reporting form are comparable to those of the WHO’s standard AEFI reporting form.^26^ Ethiopia has updated AEFI guidelines that detail the responsibilities of stakeholders, including parents/guardians, healthcare workers, district immunization officers, regional immunization officers, federal ministry of health NIP, EFDA and the national AEFI committee.^23^ Supporting the guidelines with a proclamation or regulation could increase attention towards AEFI and strengthen VP.

Despite the existence of a reporting form and AEFI guidelines, there is a gap in the distribution of these at the department level. Only a minority of the participants had copies of the reporting form and none of the departments had the guidelines. Unavailability of guidelines and appropriate forms could be one of the reasons for the low knowledge of AEFI and surveillance for AEFI. We found that most of the staff interviewed could not identify the three components of the standard AEFI case definition (an untoward/unexpected medical occurrence, follows immunization and does not necessarily have a causal relationship with vaccination). This is comparable with a study done in Albania that reported 86.3% of the participants with low levels of knowledge regarding AEFI.^27^ Low knowledge and low reporting of AEFI could also be explained by the absence of staff training on AEFI or VP. Findings from the Albania study demonstrated that the respondents with good knowledge of AEFI had training.^27^ Studies conducted in Europe also support that training is associated with a higher AEFI reporting rate among healthcare workers.^28^ To enhance appropriate response at all levels, the WHO recommends training all healthcare workers to be included in the country immunization safety surveillance system^29^ and establishing a system for feedback to communicate AEFI results.^30^ Training may also address other weaknesses identified during our readiness assessment (e.g. knowing AEFI should be reported, the flow of case reporting to the next level and improving feedback to those who report AEFI). AEFI surveillance is the mandate of the EFDA and they have been providing training as needed.

The presence of patient registers, most commonly paper based, in each department in the facilities and the inclusion of patients’ unique medical records numbers potentially facilitate the linkage of medical records across departments and vaccine registries. Information from medical and vaccination records is critical for completing AEFI reporting and investigation forms. There is no facility-wide or healthcare system–wide electronic registration or record system, which makes tracking patient encounters more challenging. For example, a child may present to a different department or facility on the following day for an adverse event after receiving a vaccine elsewhere. Therefore the only way the clinician knows about the vaccination and whether AEFI has occurred is by history taking. However, our finding revealed that the physicians do not ask children or their caregivers about recent vaccinations, and most patients do not bring their vaccination record books. This may be a contributing factor in the underdiagnosed of AEFI and low generation of data from the facilities. Paper-based systems have other drawbacks, including incompleteness of entries, illegibility, difficulties in identifying duplicate records for the same patient, storage and maintenance issues and susceptibility to damage.

DHIS2 is an open-source, web-based software platform for data collection, management, analysis and data sharing. There is also a DHIS2 mobile application that provides the same functionality as the web-based platform and works both online and offline. The WHO, United Nation Children’s Fund (UNICEF) and Gavi (the Vaccine Alliance) have partnered with DHIS2 to improve national immunization program coverage through better data collection, analysis and use.^31^ The WHO standard DHIS2 toolkit for immunization includes an AEFI tracker metadata package that facilitates the reporting of AEFI events and data collection during the investigation of an adverse event. The package includes data collection forms for facility, district and national levels and standard dashboards for analysis.^32^ The Ethiopian Ministry of Health collects vaccination administration data using DHIS2, and the staff interviewed at hospitals A and B reported familiarity with DHIS2. Adoption of the AEFI modules in Ethiopia could facilitate improved AEFI reporting from the facility to the national level. In addition, the med-safety application has been used by healthcare workers to report adverse drug reactions including AEFI. However, raising awareness of AEFI and the need for reporting will still be necessary to improve the identification of AEFI.

This project assessed the readiness of facilities to implement active AEFI surveillance; evaluated the AEFI surveillance capacities, procedures and practices at the facility; and assessed the staff’s knowledge and utilization of the AEFI protocols. One limitation of this study is the small number of facilities surveyed, as the AEFI reporting practices of three hospitals may not represent the AEFI reporting practices throughout Ethiopia. However, this evaluation was intended to assess readiness for pilot implementation of hospital-based sentinel site surveillance and variability in practices across these three facilities. Similar evaluations may need to be undertaken in other facilities being considered for implementation to ensure that activities can be adapted to each facility. In addition, we did not assess the EFDA or any intermediary levels in the reporting flow (e.g. the health centre that provides vaccine for the hospital), where AEFI are reported. AEFI surveillance involves activities such as reporting, investigation, analysis, causality assessment and communication and feedback to stakeholders and reporters. All these activities occur at multiple levels from the facility to national level, thus ensuring adequate awareness, training and resources at these levels will be important for successful implementation.

## Conclusions

Only one of three hospitals assessed in Addis Ababa in our evaluation scored >50% for readiness to implement active AEFI surveillance. The already established VP structure, guidelines, reporting form and involvement of EPHI in AEFI follow-up could be possible facilitators for the successful implementation of active surveillance. However, the readiness assessment identified areas for improvement to ensure the successful implementation of active AEFI surveillance. These included training and increased awareness of AEFI and AEFI surveillance, making AEFI guidelines and reporting forms available and ensuring a system that accommodates paper- and electronic-based records systems.

## Figures and Tables

**Table 1.** AEFI reporting in Ethiopia

Characteristics	2020	2019	2018	2017	2016	2015
AEFI reported, n	33	3	53	403	4	740
AEFI categorized as serious	11	3	1	15	4	2
Data source of reported AEFI	ND	Both EPI and NRA jointly	Both EPI and NRA jointly	Both EPI and NRA jointly	Both EPI and NRA jointly	ND
Surviving infants as per UNDP data	ND	3 455 605	3 409 051	3 160 680	ND	ND
Observed AEFI per 100 000 surviving infants	–	0.0868	2	13	–	–

ND: no data available; UNDP: United Nations Development Plan.

Source: WHO/UNICEF Joint Reporting Form on Immunization, 2015–2020.^12^

**Table 2.** Findings from the system readiness assessment, Addis Ababa, Ethiopia, February–May 2021

Proclamation	Proclamation 1112/2019^21^
Regulation	Regulation no 299/2013^20^
Pharmacovigilance structure	Product Safety Directorate (RSSID)^22^
	Pharmacovigilance Center
Reporting path	ADR e-service
	AEFI reporting form
	Mobile app (medicine safety)
	Toll-free number: 8482
Guidelines	Guidelines for Surveillance and Response to AEFI^23^
Directives	Pharmacovigilance directive
EPHI	Member of a national task force for AEFI follow-up

ADR: adverse drug reaction.

**Table 3.** Data and record system readiness for the implementation of active vaccine safety surveillance in three hospitals, Addis Ababa, Ethiopia, February–May 2021

Characteristics		Hospital A, n/N (%)^a^	Hospital B, n/N (%)^a^	Hospital C, n/N (%)^a^
Record system	Paper	5/7 (71.4)	5/7 (71.4)	6/6 (100)
	Electronic	1/7 (14.3)	0/0 (0)	0/0 (0)
	Both	1/7 (14.3)	2/7 (28.57)	–
Backup paper-based records system used		1/2 (50)	–	–
Have copies of a national guideline for surveillance of AEFI		0/5 (0)	0/5 (0)	0/4 (0)
Have copies of the AEFI reporting form		2/5 (40)	0/5 (0)	2/4 (50)
Record AEFI occurring after vaccination		Yes (only vaccination clinic)	No	No
Have the system to report AEFIs to anyone or any organization		5/6 (83.3)	1/6 (16.7)	0/5 (0)
Maintain a patient register		5/5 (100)	5/5 (100)	5/5 (100)
DHIS2 used		Yes	Yes	No
ICD-9/10 coding system used		Incompletely applied	No	No
Facility leaders review AEFI reports		Yes (only serious AEFI)^b^	No	Yes

a n/N: number of participants responding ‘yes’ (n) across assessed departments (N).

b Serious AEFI: an AEFI is considered serious if it results in death, is life-threatening, requires in-patient hospitalization or prolongation of existing hospitalization, results in persistent or significant disability/incapacity or is a congenital anomaly /birth defect.

## Data Availability

All data generated or analysed during this study are included in this article.
